# Identity Formation, Marijuana and “The Self”: A Study of Cannabis Normalization among University Students

**DOI:** 10.3389/fpsyt.2013.00160

**Published:** 2013-12-03

**Authors:** Amir Mostaghim, Andrew D. Hathaway

**Affiliations:** ^1^Department of Sociology and Anthropology, University of Guelph, Guelph, ON, Canada

**Keywords:** cannabis, marijuana, normalization, youth, identity formation

## Abstract

Over the past half-century, as use of marijuana has become more widespread in Canadian society, there are indications of a normalizing process in societal reactions and experiences of use. Among other research avenues, these trends suggest a need for further exploration of young people’s understandings of how they make the choice to use or not and how decisions relate to presentation of the self. This study draws on interviews with 30 undergraduates recruited from a larger online survey of respondents at the University of Guelph, ON, Canada. In probing their perceptions of the use of marijuana, we often found that trying/using “pot” was the default option, whereas choosing not to use required more conscious effort. With specific reference to Goffman’s contribution to a situated understanding of the self, our findings are interpreted with emphasis on further theoretical development of the normalization thesis and on the role of marijuana in identity formation among persons in the process of transition to adulthood.

## Introduction

Young people’s engagement with illicit drugs, especially cannabis, is the “single most talked about, written and broadcast about item in contemporary discourses about the state of the young” [(1), p. 12]. Research shows increasing rates of use in western countries over the last two decades among youth and adults (2–4). Observance of this trend in the United Kingdom led Parker and his colleagues to initiate a discussion about the normalization of illicit substance use. Not only has the use of drugs in certain contexts increased, but socio-cultural attitudes regarding use, they argue, have shifted “from the margins toward the center of youth culture” [(1), p. 152]. The use of marijuana in particular no longer can be described as marginal or deviant in the sense of denoting membership in a distinctive subculture (5).

Cannabis is the most widely used illicit drug in western nations. Estimates in Canada suggest that almost half of the population over age 15 has used cannabis at least once, and more than half of university undergraduate students (6, 7). Starting in the 1990s, Adlaf and his colleagues have documented increasing prevalence and incidence of use in all age cohorts, with estimated lifetime use among Canadians increasing from 23% in 1989, to 28% in 1994, to 44% in 2004. With respect to prevalence, lifetime use alone may be less indicative of normalization than increases in recent or regular use. Studies in the UK suggest that 10–15% of late adolescents are recent, regular recreational cannabis users, with this proportion rising to 20–25% among young adults (3). Similarly, young Canadians are not only more likely than the previous generation to have used the drug in their life time; they are more likely to have done so within the past 12 months. About 70% of those from 18 to 24 years old reported using cannabis at least once. And nearly half of those between 18 and 19 reported use in the past-year, a number that has doubled since 1994 (6). Marijuana use is therefore common among students, and most say they find it “easy” or “very easy” to obtain (8).

A recent European study traces attitudes of youth between 14 and 19 years of age (9). Over this period it documents a change in their opinions from being negative and skeptical to positive and accepting of the use of marijuana. This is not to say, the authors note, that use is normal in the sense that everybody uses, but normal in the sense that use is ordinarily perceived as legitimate by users and non-users alike. Despite continuation of the ban on marijuana, the stigma attached to users is increasingly connected to the context of consumption as opposed to use *per se* (10). Whereas “reefer madness” era claims are frequently rejected, concerns regarding health, and social risks still remain (11, 12).

A fuller understanding of what is meant by normalization requires attention to not only rates of substance use and availability, but also to more abstract socio-cultural dimensions of accommodation by non-users of the drug (3). Thus, further qualitative research is needed to attain “a more nuanced appreciation of how the boundaries of morality – and of deviance and problematic use – are defined, and how these definitions vary over time, context and social identities” [(13), p. 144]. To better understand the role that marijuana use plays in the identities of emerging young adults, this paper draws on interviews conducted in a study of undergraduate students at a Canadian university. The normalization process, we will argue in particular, is facilitated by a fluid view of self in which the identity of “user” and “non-user” is not fixed, but rather more contingent on the situated context or social circumstances of marijuana use. To shed more light on features of the contemporary context, and the social meaning of marijuana use, this paper draws on Goffman’s understanding of identity as a situated construct which is flexible itself.

## Materials and Methods

A small pilot grant to run the study was provided by the College of Social and Applied Human Sciences at the University of Guelph. The study protocol was developed and approved through consultation with the University Research Ethics Board. Interview participants were recruited in the Winter of 2010 from a larger online survey of students enrolled in the second author’s course on introductory criminology. This course has a diverse cross-section of undergraduates from a variety of programs who take the first-year class as an elective. In addition to informed consent, the online questionnaire included an invitation to participants to take part in a face-to-face follow up interview, with instructions to provide their contact information or contact the first author by telephone or email.

### Sample characteristics

Between January 2010 and May 2010, 130 participants from a total class enrollment of about 388 students (33.5%) completed the voluntary online survey. Nearly two-in-three (63%) of the participants were female. Their ages ranged from 18 to 25 years old, though most were younger first-year students as reflected by the mean age of the sample (18.5 years). Although participants came from variety of ethnic backgrounds, the majority (80%) identified as white or Caucasian. Only five were born outside of Canada, and only four from provinces other than Ontario. Of those who responded to a family income measure, about half reported household income in the high range (more than $75,000), as compared to one-in-five who reported lower income ($15–35K) and about one-third who reported middle income ($35–50K). Forty percent reported financial support for their schooling came mainly from parents. One-in-three supported themselves through part-time work, 15% through full-time work, and 14% reported that their primary support came from student scholarships or loans. Three-quarters of respondents were pursuing a degree in social science or humanities, as compared to one-in-four reporting that their programs were in engineering or the natural sciences.

Sixty percent of the participants had used marijuana, the vast majority of whom (90%) reported having used it for the first time during high school, sometime between 15 and 17 years of age. Three-quarters of those students who had ever used it reported having done so in the past 12 months. Two-thirds of past-year users used it once a week or more. The majority (∼80%) of those who had used in the past-year reported using less than a standard “joint” (about 1/2 g of cannabis) on a typical occasion. Most respondents had not purchased marijuana for themselves during the previous month, yet found it “very easy” or “easy” to obtain. One quarter had spent less than $50 buying cannabis; only six reported spending more than $100. By contrast nearly half of past-years users indicated that it was gifted, or that friends had shared it with them. Given the high ratio of females in the sample, this finding may reflect the observation in prior studies that women in particular report they need not always purchase their own cannabis to use it regularly [see (5)]. For respondents who had not used or were no longer using cannabis, nonetheless financial costs were cited second only to work/school obligations as the most important reason for abstaining.

With regard to other drug use, nearly all who took the survey had used alcohol at least once during the past month, and about one-third had smoked tobacco. Use of other drugs was more infrequent or sporadic, with past-year use of mushrooms (*n* = 16) and ecstasy (*n* = 11) the only other substance use reported by more than one-in-ten. Survey items about attitudes toward different substances show that the majority of users and non-users of cannabis consider it less dangerous than other types of drugs – including legal substances like alcohol and tobacco. When asked about the differences, apart from use *per se*, between marijuana users and non-users, more than half of both groups said no differences at all. These survey findings indicate consistency in attitudes concerning marijuana that appear to be consistent with socio-cultural dimensions of the normalization thesis.

### In-depth interviews with students

Quota sampling with respect to gender, age, and drug status was used to select 30 interview participants 18–21 years old. To attain sufficient representation of non-using as well as marijuana using students, we also sought to ensure that at least one-third of participants were either non-users or former users [see (3)]. Interview participants were each paid $20 to acknowledge their time and contribution to the study. All interviews took place in a private campus office and were digitally recorded to be transcribed verbatim.

Questions in our interview schedule were informed by sensitizing concepts from a variety of sources including work by Jenkins (14) and Hammersley et al. (13) on social identity and marijuana use. Related central themes in the analysis that follows pertain to self-perceptions, or “significations,” and to different forms of “categorization” and “negotiation” of the boundaries observed. For present purposes in this paper, questions from the interviews and related probes of interest focus on young people’s recollections of the process through which they made the choice to use or not use marijuana, and how they understand its role or meaning in relation to their own identity as a user or non-user.

A cross-case analysis was done during transcription, using the constant comparative method, to evaluate the qualitative data for emerging themes or concepts by seeking out disconfirming evidence (15). Rather than imposing patterns, themes, and categories, these were allowed to emerge from the data. Discovering relationships between the categories began with analysis of initial observations and continued throughout the research process, by continuous refinement of the category coding. The meaning of the category thus evolved with the research as rules of inclusions and exclusion were changed to fit the data (16). The method of constant comparison enables new topological dimensions and relationships to be discovered (17). Through this iterative process, we sought to better understand the attitudes of users and non-users in our study in terms of the extent to which they may converge or differ, and further implications of the role of marijuana for identity formation among undergraduate students.

## Results

### Converging attitudes of users and non-users

Converging attitudes of marijuana users and non-users about the social status of the drug were often documented by student’s common recognition that the exaggerated claims of the Reefer Madness era are unwarranted, deceiving, often humorous, and foreign to the lived experience of the majority of students. One non-user, for example, said that marijuana use “won’t kill anyone or make them drop out of school but, you know, it is just not for me. I just don’t like doing it.”

Notwithstanding mixed opinion on the potential risks and harms, accommodating attitudes of non-users ordinarily were asserted on the basis of a person’s right to choose. One non-user stated: “It’s their life, it’s their choice; none of my business if they are hurting themselves.” Another student similarly observed:

“Skateboarding is dangerous; it is fun but dangerous and not everyone can do it. I love doing it and it is no one else’s business that I might hurt myself. It is the same thing with pot. I don’t like it and I think it’s bad for you, but who am I to judge anyone? If they want to risk their health, well let them do it” (21-year-old male).

Comparing it to alcohol, one non-user said: “I love to have a few drinks and get drunk, so who am I to tell people not to smoke pot? … as long as people are having fun, they should do what gets them in the mood to party and have a good time. After all you see pot as often as alcohol these days.” Indeed it was routinely observed that marijuana use within their peer groups was so common, and taken for granted among university students, that the questions seemed surprising or perhaps naïve to some. To illustrate, when asked if she would have a problem with the presence of marijuana at a party, one non-user replied: “If there are parties without at least some pot, I have never seen one of those!”

Despite its evident ubiquity at large parties among students, use in smaller gatherings or social situations is far from unequivocally accepted by non-users. For example:

“It’s not like I don’t like it you know … as I said, it’s cool to have it around at a party, but I don’t like it if I am just there to play some video games or whatever” (22-year-old male).

“I really don’t mind if people are using it, like if I am with friends and they are going to get high that’s cool, but I just don’t want to be around when they are doing it. When they are actually smoking, I might go out for a walk or go on my cell, you know, just till they’re done” (19-year-old female).

Only one non-user in our sample indicated that he avoided socializing with his peers when they are “stoned.” He said: “When my friends get stoned, they get very weird … it’s like they’re in a different world. They’re just not like usual, so I don’t have a good time when they do it. Maybe because I am sober you know, but well they try to not smoke or get stoned when I am around.” More commonly, objections from non-users had less to do with users “getting high” than fear of sanctions from authorities and potential health risks due to smoking marijuana. Some users had these types of fears and sentiments as well. For example:

“First of all I don’t like smoke, so I just don’t like to be around when people are smoking anything. But with pot, I just feel like what if cops come in? What if they get caught? Then I am in trouble too … they can go get high, come back, and have a good time; chances are I am having a drink at the time as well” (20-year-old non-using female).

“I would leave if anyone smokes anything in a room. It is against residence policy and I don’t want to be charged with something I am not doing. I couldn’t afford the ticket and what if I get kicked out? It’s just not worth it” (19-year-old female, former user).

“… I hate smoke. I mean I don’t care if my friends are high when I see them, but if we are inside and they are smoking, I am out of there. It makes me cough up a lung! As I said, I love pot brownies. But I don’t smoke it because it’s horrible for you” (20-year-old male).

Further to these caveats and boundaries observed in social interactions between users and non-users, converging attitudes between the groups were also demonstrated in their common understandings of problematic use. Many students shared the view that marijuana use in moderation – or even heavy use – is safe, assuming that the user is still “taking care of business.” One user suggested, “it’s not like it’s alcohol, you know? You can still get stuff done … problematic use is when you can’t get your stuff done.” A non-user stated: “I didn’t know you could smoke too much pot. I guess if you are not missing class or work, it’s not too much. I mean my boyfriend is high all the time, and he seems fine.”

To further illustrate this view of marijuana as benign, at least compared to alcohol or other substance use, respondents commonly suggested that excessive use is not determined necessarily according to use levels or amounts; it is rather more contingent on the experience of use. “It is not like drinking,” for example, said one user; “someone can be stoned all the time and not have a problem, and someone can smoke once a week and even that might be too much for them.” Another one observed: “There are no such things as rules of thumb when it comes to quantity of marijuana you use. It is really up to the person, as long as you can get stuff done.”

To better understand the distinctions that are made between “users” and “non-users” among students in both groups, we asked about the differences that they saw between groups beyond the use of cannabis itself. Reported differences were viewed as negligible by most, though some observed that users are perhaps more “open-minded,” open to new things, or “easy going” than non-users. For example: “I don’t think there are much differences … people who use are I guess more open-minded about things. They are more likely to try new things” (19-year-old female user). And another: “I don’t see much of a difference … My friends who use it seem to be a little bit more easy going than the rest I guess, but there is no telling” (20-year-old male non-user).

Despite the lack of clear distinction between users and non-users, social censure is still evident in both groups. However, stigmatizing labels are reserved for noted “pot heads,” or those who abuse it, as opposed to the more typical representation of the marijuana user who uses it responsibly with no adverse effects. Most notably, we found that some student’s designation or perception of the very existence of a “user” appears to be more fluid than the term tends to suggest. One user stated, for example: “I really don’t think someone is a user … what I consider to be a marijuana user are really pot heads, you know people who use it way too much” (20-year-old male). And a non-user said:

“Do I think there is such a thing as a marijuana user? Of course, but I mean they are users because they use the drug. But really I consider marijuana user as someone who abuses it. I sometimes smoke a cigarette; that doesn’t make me a ‘smoker.’ The same way, if someone is smoking pot at parties, they are not marijuana users …” (19-year-old female).

Another female student also made the point: “Simply because I play intramural soccer; that does not mean that I am a soccer player. So if I am smoking pot sometimes, I am not a ‘pot smoker.’ I just use it sometimes.” The identity of “marijuana user” from this standpoint appears to be much like a “hat” or article of clothing that young people wear if, when and where they make the choice to use [see also (10)].

### Marijuana and identity

Because responsibilities and social roles may vary, it appears “the fit” is not the same for every “user.” What is the role of marijuana use in identity formation? How and why do some young people choose to use cannabis? And why do others choose not to? For some of our respondents, as in the following examples, the primary reasons for abstaining were related to social obligations, prior commitments, or their responsibilities to family or to work:

“My grandmother promised that if I do not use drugs during high school she will give me a $5,000 gift to pay for my first-year tuition at the university. I mean that is a big incentive and that meant I can use my money to go traveling, so to me it just made sense to not smoke pot or use any kind of drug … of course I was tempted and still might try it in the future, but five grand is a lot of money” (19-year-old female).

“My boyfriend and I made a pact that we will not use pot. It was just a promise that we gave each other. I don’t know if he has kept it, but I always looked at it as something that made us different than other couples” (19-year-old female).

“I guess I didn’t start because there was no opportunity to start. I had to study and take care of our family shop, so I just had no time to go to parties to even get close to someone who has pot. Now in school it is the same thing, work work work. I just can’t get a break to even have a beer, never mind a joint!” (21-year-old male).

By contrast, most non-users cited reasons for abstaining that appeared less practical than meaningfully symbolic, relating to perceptions of identity or status and their presentation of the self. Some of them made it clear that they intended to maintain their images as “clean-cut” and hard-working students. And some had aspirations, or positions to uphold, requiring them to set themselves apart from others by example. One student said: “I didn’t smoke pot because I was the student union president at our high school. I wanted to be the clean-cut guy who people look at as responsible. Not like pot users cannot be responsible but it is the image that matters, you know?” (20-year-old male).

More generally non-users said they wanted to convey an image that is different from other mainstream youth in the sense that abstinence is the new form of rebellion, since using marijuana is increasingly the norm. For example:

“I’ve never smoked pot because well it seemed like a commonplace thing to do. It seemed like it was just another thing that everyone was doing. I am a rebellious person but smoking pot seemed more conformist than rebellious” (20-year-old male).

“I really didn’t want to be like everyone else. You couldn’t swing a cat and not hit a pot user in my high school. They were just everywhere and you couldn’t get away from them all, so I made a point of not using. What’s the point of being like everyone else?” (19-year-old male).

“I have always wanted to be myself; hence why I dress this way [in a style that was unique to say the least]. I have always wanted to be the girl who is different, who is not like everyone else. I think if no one smoked pot I would have been the biggest pot head” (20-year-old female).

Students’ recollections of how they became a “user” converged with the perceptions of non-users that marijuana use is normal among young people that they know. Indeed, while abstinence apparently required more conscious effort, the choice to use for many seemed to be taken by default. These examples indicate that marijuana use by students can convey a wide variety of meanings, from mundane or “commonplace” to intimately connected to a sense of independence for emerging young adults:

“I don’t know why I first started smoking. I guess it was just something to do. Everyone else was doing it and it seemed harmless” (21-year-old male).

“Well a lot of people I hung around with used it. I mean it’s like having a drink or taking a puff off the cigarette. You give it a try to see how things go” (19-year-old female).

“It wasn’t really a decision but sort of like a … rite of passage … it was like you are not a kid anymore now that you have smoked pot” (19-year-old female).

In the situated context of attending university, we encountered differing perspectives on the matter of opportunities provided for using marijuana. As noted previously, some students found that work and school commitments restricted their free time and freedom to use. Notwithstanding these experiences, it is clear that many others find life in university gives ample opportunity. One male, aged 20, said: “My pot smoking has increased now. I don’t have to work. I don’t have to really do anything but study, so it leaves a ton of time to go out and party and get stoned.” Another male student, age 20, reported: “I never smoked pot in high school … now in university, it is different. I have lots of time to myself and less responsibility than before, so I have started smoking some when I go out with friends.”

The majority of users in our study said attending university afforded more opportunities and freedom for using marijuana whether they had used it or never tried before. The greater freedom they reported was commonly attributed to the anonymity afforded by the university environment and community. The transition from high school to university often means moving from a small town to a bigger place. Accordingly, a 19-year-old male stated that: “I loved the fact that I could smoke pot and still keep it a secret. Do you know how hard it is to keep something a secret when you have only 20 people in your high school graduating class? If I had even been close to a joint my parents would have found out quite quickly.”

Marijuana use, for some, appears to be facilitated by being better able to blend into the environment. For others, it is more of an expression of being able to define themselves in ways they never thought they could before. This point is illustrated in these final two examples of how students understand their use of marijuana in relation to their presentation of the self:

“I didn’t smoke in high school because we lived in a small town north of Guelph. Everyone knew everyone, so if I smoked one joint then everyone would know. I really wanted to get into university and needed good letters and volunteer work and I knew that wouldn’t have been possible if people knew I smoked pot, so I didn’t. When I came here though, it seemed like you could get lost in the sea of students and no one would be any wiser. I could smoke all the pot that I wanted and still manage to show a face of a clean-cut girl. I just loved the anonymity” (20-year-old female).

“I liked university; you could distinguish yourself in other ways. Like in high school we couldn’t even wear our uniforms the way we wanted to. But once I came to university, my image could have been more than just that girl who doesn’t smoke pot. I could define myself uniquely in a million different ways. Pot became a non-issue then” (19-year-old female).

## Discussion

There are several limitations of this study, so some caution is needed in interpreting results. Our two-stage process of recruitment based on an online student survey of respondents in an introductory crime class relies on self-selection and is not truly representative of university students, nor even students taking introductory criminology. We successfully recruited only one-in-three potential respondents in the class to complete the online survey. Another study underway at the same university and other universities in Canada confirms that small incentives (such as a $20 payment or 2% participation mark for volunteering) dramatically improve upon response rates in such studies. When a survey includes questions about illegal conduct, a high degree of non-response is typically expected. Yet levels of participation in Canadian drug studies are within the standards expected of most surveys (6), and self-reports on drug use have been shown to be quite valid (18). The trend toward greater acceptance of cannabis may also mitigate to some extent reluctance among users to participate in interviews and surveys (19).

Based on in-depth interviews with “users” and “non-users,” we have explored how they identify the practice and how their attitudes relate to normalization of marijuana use. An important aspect of marijuana normalization is less concerned with how users perceive their use as “normal” than how it is regarded by others in society, regardless of whether they approve. Marijuana use today still carries a certain stigma, the management of which requires the user to observe boundaries and have rules about negotiating conflict (11). However, attitudes of users and non-users are converging (13, 20). The prevalence of marijuana use by young adults, and the converging attitudes of users and non-users, means we can no longer speak of users as belonging to an identifiable deviant “subculture.” Rather use communicates a style that might be viewed as “conventionally unconventional” (21) by some youth and merely conventional, conformist, or commonplace by others. Indeed, the very notion of a “user,” or non-user, has different connotations in this normalizing context.

Taking an opportunistic “puff” at a party more often signifies commitment to having a good time, or “fitting in,” than a clear intention or desire to “use.” This may be more apparent in the younger generation, but it appears that attitudes among the “over thirties” are also becoming more liberal (1, 22, 23). Marijuana use in certain settings is likely largely understood by young people as it is by many middle-aged adults. However, use by youth appears to be less ritualistic or confined to certain settings, Zinberg (24) argued; that is, young people tend to be more flexible in their use (1, 3, 25). Similarly Parker and his colleagues found that students rarely gather for the purpose of smoking marijuana. It was more often used as a complement to other activities like drinking, playing video games, or simply “hanging out.”

Howard Parker situates the normalization thesis in scholarly discussions about changes in the process of transition from adolescence to adulthood. In today’s “post-modern world’ youths” attitudes, opinions, and their use of leisure-time all are being shaped in different ways than those of preceding generations (26, 27). The process of becoming an adult, it is argued, is fundamentally different for contemporary youth in the formative period of post-adolescence (28). The stage(s) between childhood, adolescence, and adulthood are not only longer, but more complex than in previous generations (29). Changes in the journey to adulthood, in particular, are reshaping the nature of leisure and pleasure in a way that is specific to the post-modern world (1, 30). As leisure and consumption replace work and production as the main source of identity formation (2), young people form and maintain an idea of the “self” that “expresses its integrity through parading its identity” [(31), p. 882]. Among other implications of a changing workforce and associated changes in the school to work transition, youth today have more time to participate in leisure and shape their identities through leisure-time consumption.

Through consumption young people not only shape their leisure-time; they also shape formation of their own identity. “The relationship between consumption and identity formation is one compelling explanation for why drug use has become more common” [(2), p. 443]. With increasing numbers of “ordinary” young people growing up “drug wise” and accepting of controlled or “sensible” drug use (3, 32), the recreational use of marijuana has become part of their leisure repertoire, or just another aspect of the consumer lifestyle (1). As “time out” becomes commoditized (33), the use of certain substances, much like fashion, is becoming just another form of “symbolic consumption” that conveys meanings about self, identity, and status [cf. (34–39)]. Further, notwithstanding the limitations of this study, our interpretation of the findings indicates that a more nuanced understanding of the social context of normalization among emerging young adults calls for a more flexible or fluid interpretation of identity with particular attention to the situated “self.”

### Conceptualizing the post-modern, fully situated self

For Plato, the “reality” we think we see is really more like shadows cast upon the wall of a cave by the flickering light of the campfire. What we imagine we are seeing is but a representation of something that exists only in our minds. Likewise, the post-modern self, in contrast to the modern which seeks a sense of order or enduring essence, is premised on rejection of an essence altogether. The post-modern self, accordingly, consists of images (not essences) which are part of relationships – not of the individual (40). Thus, the self is only real within its social context; it is wholly interactive, existing only in the interplay of images with no underlying signifiers, or essence of its own [(41); see also (42)].

Park (43) was the first sociologist to conceptualize individuals as actors who are “seeking recognition.” Our need for social recognition and acceptance by our peers compels us to present ourselves to others in a way that we believe will be acceptable to them. This “mask” becomes “our truer self, the self we would like to be” (p. 739); hence Park recognizes the fluidity of the self while assuming a “true self” exists. Goffman’s dramaturgical perspective calls for further “critical recognition of the conventionalizing influence of the social looking-glass” [(44), p. 277]. Goffman’s self is an entirely “social self” who is either performing or preparing to perform for a particular audience. In his words, thus “while people usually are what they appear to be, such appearances [are] managed” (1959, p. 77). This view of self is not entirely dependent on the actor, but rather it develops as a result of interaction between the actor and the audience.

In his theatrical analogy, Goffman’s front stage is comprised mainly of the setting and actor’s personal characteristics. The setting is the physical environment; for example, marijuana smoking in parties of mixed attendance typically occurs away from other guests in a different room, or outside (13) – i.e., in the backyard, on the balcony or porch, or partakers might go for a walk around the block. Thus, successful participation in a particular setting requires the user to be familiar with “regional behaviors” that dictate the boundaries of social accommodation (45). The actor’s personal front stage comprises items that are identifiable by the audience as part of the performance. Much like the sword of the sword fighter, possession of a “joint” is part of the personal front stage of the marijuana user. And much like the sword fighter can also be a basketball player when s/he is in possession of a ball, a nightly marijuana smoker might be in the position of sending citizens to jail for the same behavior garbed in a judge’s robe the following morning. As marijuana use has shifted from a marginalized behavior to one considered normal within a certain context, we cannot view the “user” as a homogenous (id)entity; and we can no longer look at settings as specific to “users” (24).

Whereas manner and appearances need to be consistent or harmonious in a given setting, this consistency only needs to last as long as the front stage itself does. Our judge who must uphold the law in court may also be in favor of cannabis reform, and advocate for changes in the law in different roles on the job and as a private citizen. The front is not created by the actor *per se*; rather it is chosen from a repertoire of selves to be consistent with the setting or the situation. Providing a convincing front requires not only choosing the proper schema but also effective and consistent communication of the characteristics of the chosen role (46). There are also tactics for concealing certain secrets, such as occasional drug use, that are not in harmony with the intended performance. Hidden aspects of the front stage are present in the “back stage.” Since the audience should not be aware of this deception, the actor must employ techniques to make sure that the secrets of the back stage do not leak to the front stage, which would ultimately discredit the performance.

Goffman’s most important theoretical contribution is replacing the “deep value” with the “face value” by challenging the distinction between “real” and “staged” (40). His conceptualization of the self marks the transition from representations to simulations where signs are no longer real on their own but dependent on other signs within a reproduced reality (47). This wholly situated self blurs the lines between real and imaginary to the point that the distinction becomes one of style rather than substance. Goffman argues that a person is made up of multiple, loosely connected selves [(48), p. xlviii], as opposed to an essential self. The transition from a “modern” to “post-modern” view of self is most notably consistent with revisions Goffman (49) made in the second edition of The Presentation of Self in Everyday Life. Whereas the first edition conjures up a cynical vision of the world where actors manipulate their audience by hiding their real selves behind the mask of the front stage, in the later version manipulation and deception are recast as mutually accepted and expected social roles or representations played out between an actor and her audience.

Most significantly, Goffman cautions readers against taking the dramaturgical metaphor too literally. Unlike a stage performance, where actors may remove their masks after the curtain call, beneath the mask is not a face but a repertoire of other masks chosen to suit the social role, setting, or situation (50). Whilst he distinguishes between the “public” and the “private,” this is not to suggest a “true private” and “false public” (51, 52). Nor is it captured by the term “impression management” which highlights the distinction between appearance and reality, wherein appearance is “manipulative” and reality is “honest” (53–56). For Goffman, representation is an end unto itself. His situated self is neither manipulative nor honest; it is not real or imaginary – rather it is fluid. In Platonic terms, it is a shadow with no person. Similarly, Goffman echoes Horace when he says “we are dust and shadows” (pulvis et umbra sumus). A more nuanced understanding of the normalization process, with respect to cannabis and other substance use, would benefit from further theoretical engagement with Goffman’s situated understanding of the self.

## Conflict of Interest Statement

The authors declare that the research was conducted in the absence of any commercial or financial relationships that could be construed as a potential conflict of interest.
